# Transcript Profiling Identifies Dynamic Gene Expression Patterns and an Important Role for Nrf2/Keap1 Pathway in the Developing Mouse Esophagus

**DOI:** 10.1371/journal.pone.0036504

**Published:** 2012-05-02

**Authors:** Hao Chen, Jianying Li, Haiyan Li, Yuhui Hu, Whitney Tevebaugh, Masayuki Yamamoto, Jianwen Que, Xiaoxin Chen

**Affiliations:** 1 Cancer Research Program, JLC-BBRI, North Carolina Central University, Durham, North Carolina, United States of America; 2 Frontier Bioinformatics Solution, LLC, Cary, North Carolina, United States of America; 3 Center for Musculoskeletal Research, University of Rochester, Rochester, New York, United States of America; 4 Department of Medical Biochemistry, Tohoku University Graduate School of Medicine, Sendai, Japan; 5 Department of Biomedical Genetics, University of Rochester, Rochester, New York, United States of America; 6 Division of Gastroenterology and Hepatology, Center of Esophageal Disease and Swallowing, University of North Carolina at Chapel Hill, Chapel Hill, North Carolina, United States of America; Cincinnati Children's Hospital Medical Center, United States of America

## Abstract

**Background and Aims:**

Morphological changes during human and mouse esophageal development have been well characterized. However, changes at the molecular level in the course of esophageal morphogenesis remain unclear. This study aims to globally profile critical genes and signaling pathways during the development of mouse esophagus. By using microarray analysis this study also aims to determine how the Nrf2/Keap1 pathway regulates the morphogenesis of the esophageal epithelium.

**Methods:**

Gene expression microarrays were used to survey gene expression in the esophagus at three critical phases: specification, metaplasia and maturation. The esophagi were isolated from wild-type, *Nrf2^−/−^*, *Keap1^−/−^*, or *Nrf2^−/−^Keap1^−/−^* embryos or young adult mice. Array data were statistically analyzed for differentially expressed genes and pathways. Histochemical and immunohistochemical staining were used to verify potential involvement of the Wnt pathway, Pparβ/δ and the PI3K/Akt pathway in the development of esophageal epithelium.

**Results:**

Dynamic gene expression patterns accompanied the morphological changes of the developing esophagus at critical phases. Particularly, the Nrf2/Keap1 pathway had a baseline activity in the metaplasia phase and was further activated in the maturation phase. The Wnt pathway was active early and became inactive later in the metaplasia phase. In addition, *Keap1^−/−^* mice showed increased expression of *Nrf2* downstream targets and genes involved in keratinization. Microarray and immunostaining data also suggested that esophageal hyperkeratosis in the *Keap1^−/−^* mice was due to activation of Pparβ/δ and the PI3K/Akt pathway.

**Conclusions:**

Morphological changes of the esophageal epithelium are associated with dynamic changes in gene expression. Nrf2/Keap1 pathway activity is required for maturation of mouse esophageal epithelium.

## Introduction

Morphological changes in developing human organs require coordinated activation of gene transcription and signaling pathways [1]. The epithelial cells lining the human esophagus transform from simple columnar into ciliated epithelium at an early phase. The ciliated epithelium is then gradually replaced by a squamous epithelium until a non-keratinized stratified squamous epithelium. Morphological changes during human esophageal development have been well-characterized for several decades [2], [3]. However, the molecular mechanisms underlying these morphological changes remain largely unknown.

Studies using mouse genetic models provided initial insights into the roles of transcription factors and signaling pathways for the morphogenesis of the esophagus [4], [5]. The esophagus is specified from the foregut tube at embryonic day E9.5 in mice, and at four weeks in humans. In mouse embryo, the esophagus is completely separated from the trachea at E11.5. Mutation of genes encoding transcription factors (e.g., *Sox2* and *Ttf1*) and signaling molecules (e.g., *Noggin*, *Shh*) disrupts the separation process, leading to the formation of esophageal atresia [6]. From E11.5 to E15.5 the esophageal epithelium is transformed from a simple columnar epithelium to a multiple-layered epithelium. Towards the end of this phase the epithelium starts to lose columnar cell differentiation markers and express squamous cell markers. From E15.5 to birth, columnar features are almost lost and the epithelium is further stratified. From postnatal day 7 (P7) onwards, the top layer of the stratified squamous epithelium starts the enucleating process and forms a keratin layer which is not present in the human esophagus [5], [7], [8], [9].

According to these morphological changes, the development of the esophagus can be divided into three phases: specification phase (E9.5–11.5), metaplasia phase (E11.5-P7), and maturation phase (P7-adult). Our previous studies showed that Bmp signaling plays a two-stage role in the developing esophagus [4]. During the early metaplasia phase (E11.5–15.5), the Bmp pathway is inhibited by *Noggin* to allow stratification to occur. Subsequently, the Bmp pathway must be activated to promote squamous differentiation of the top layers of the stratified epithelium [4]. Interestingly, other signaling pathways including Wnt pathway and Shh pathway are active in the separating esophagus at the early specification phase (reviewed by Morrisey and Hogan [10]). Nevertheless, it is unknown whether these signaling molecules assume a dynamic change of expression pattern similar to Bmps.

In our previous study on human Barrett's esophagus, a metaplastic condition in which the stratified squamous epithelium of the lower esophagus is replaced by intestinalized columnar epithelium, we found that several transcription factors such as *Nrf2* (nuclear factor erythroid derived 2 like 2, or *Nfe2l2*) and small Maf proteins (MafF, MafG) were enriched in the normal human esophagus as compared with Barrett's esophagus [11]. As a major cellular defense pathway, the Nrf2/Keap1 (kelch-like ECH-associated protein 1) pathway is known to regulate expression of enzymes involved in detoxification and anti-oxidative stress response [12]. Nrf2 forms heterodimers with small Maf proteins and binds to the antioxidant-responsive elements of target genes when cells are exposed to oxidative stress or xenobiotics. Keap1 regulates the function of Nrf2 by retaining Nrf2 in the cytoplasm under normal physiological conditions, and by allowing nuclear translocation of Nrf2 under stress conditions. Certain *Keap1* mutants have a dominant-negative effect on wild-type *Keap1* [13]. In addition to its function in stress response, the Nrf2/Keap1 pathway is known to participate in wound healing, inflammation resolution, apoptosis, and keratinocyte differentiation [14].

*Nrf2* ^−/−^ mice developed normally. *Keap1* ^−/−^ mice died within three weeks after birth, probably due to malnutrition as a result of hyperkeratosis in the esophagus and forestomach. In the esophageal epithelium, *Keap1* ^−/−^ mice expressed higher levels of *Krt1*, *Krt6* and *Lor* and lower levels of *Krt13* and *Inv* than the wild-type mice. These phenotypes were due to superactivation of *Nrf2* with the help of small Maf proteins because both *Nrf2* ^−/−^ *Keap1* ^−/−^ and *MafF*:*MafG*:*Keap1* ^−/−^ rescued the *Keap1* ^−/−^ phenotype [15], [16]. These studies clearly indicate that the Nrf2/Keap1 pathway plays a critical role in the development of esophageal epithelium.

In this study, we examined gene expression in the esophagi of wild-type and mutant mice (*Nrf2* ^−/−^, *Keap1* ^−/−^ and *Nrf2* ^−/−^ *Keap1* ^−/−^) using gene microarrays. Our goal was to survey gene expression during the development of mouse esophageal epithelium, and to better understand the role of the Nrf2/Keap1 pathway in the process.

## Materials and Methods

### Animals

Wild-type C57BL/6J mice were purchased from the Jackson Laboratory (Bar Harbor, ME). *Nrf2* ^−/−^ and *Keap1* ^+/−^ mice on C57BL background were obtained from the Experimental Animal Division, RIKEN Biosource Center (Tsukuba, Japan) [15]. BAT-GAL and TOP-GAL mouse lines were purchased from the Jackson Laboratory, and they were maintained on C57BL/6 and CD1 background, respectively [17], [18].

**Figure 1:**
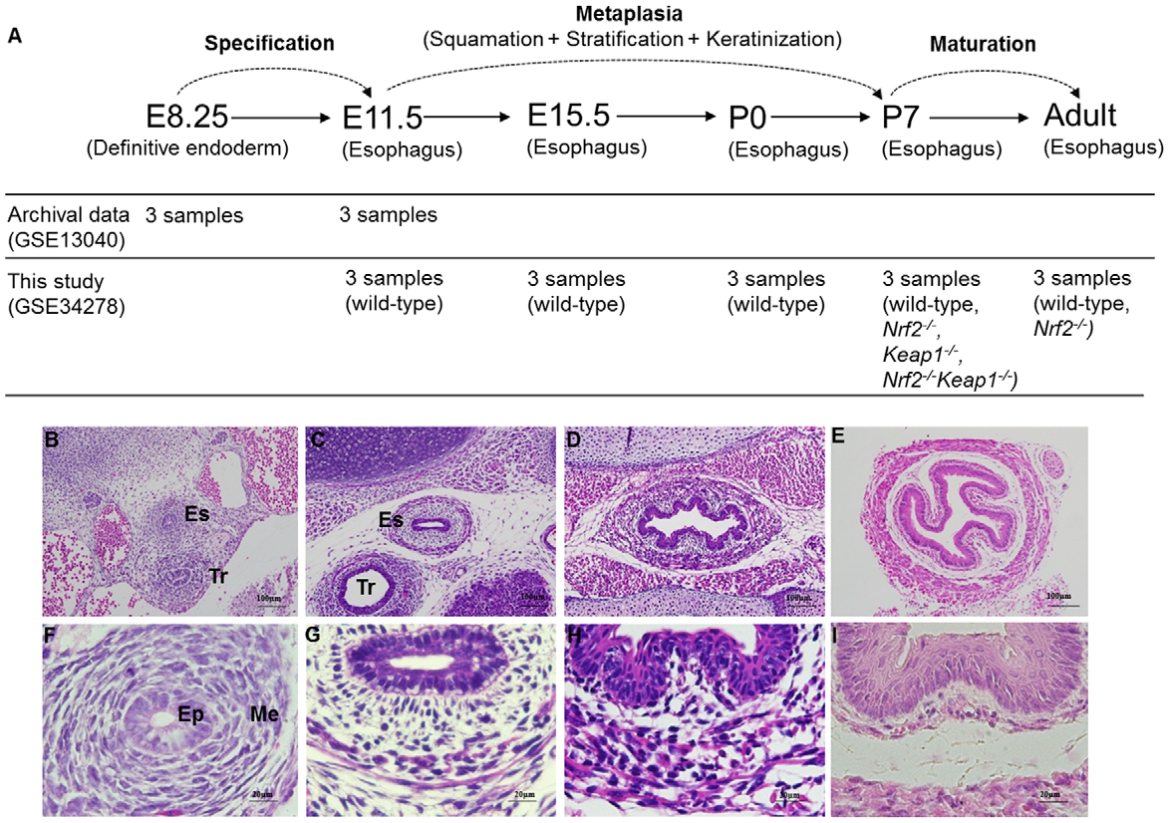
Changes in histology during mouse esophageal development and sampling scheme in this study. (A) Three esophageal samples in each group at each time point were used for analysis. (B–I) H&E staining of paraffin sections of mouse esophagus showed histological changes of esophageal epithelium and mesenchyme in metaplasia phase (E11.5, E15.5, P0 and P7). Panel F, G, H and I (size bar = 20 µm) are magnifications of Panel B, C, D and E (size bar = 100 µm), respectively. Es, esophagus; Tr, trachea; Ep, epithelium; Me, mesenchyme.

These mice were bred in-house to generate embryos and offspring with proper genotypes. Mice were PCR-genotyped according to protocols provided by the original developers. Esophagi of E11.5, E15.5, P0, P7, and adult (8 weeks old) mice were dissected and snap-frozen for future extraction of total RNA. Part of each esophagus was fixed in 10% buffered formalin or frozen for future use in histology. Three esophageal samples from each group at each time point were harvested. The following tissue samples were harvested for gene expression profiling: (1) wild-type esophagi at E11.5, E15.5, P0, P7; (2) wild-type, *Nrf2* ^−/−^, *Keap1* ^−/−^ and *Nrf2* ^−/−^ *Keap1* ^−/−^ esophagi at P7; (3) wild-type and *Nrf2* ^−/−^ adult esophageal epithelium (see Figure 1A for the sampling scheme). All animal experiments were approved by the Institutional Animal Care and Use Committees (IACUC) at the University of Rochester and North Carolina Central University (protocol number XC-12-03-2008).

### RNA isolation and quality check

Total RNA was extracted from individual mouse esophagi (E11.5, E15.5, P0, P7 and adult) with an RNeasy Fibrous Tissue Mini Kit (Qiagen; Valencia, CA). These RNA samples were checked for their quality using gel electrophoresis, and their concentrations were measured using spectrophotometry. Their quality (RIN>7) was further checked with Bioanalyzer (Agilent Technologies; Santa Clara, CA) at the Genomics Core Facility, Lineberger Comprehensive Cancer Center, University of North Carolina at Chapel Hill.

### Microarray data collection, data pre-processing and probe annotation

Microarray experiments were performed at the Genomics Core Facility with Agilent two-channel mouse 4×44k microarrays. Red channel (Cy5) was used for esophageal samples, and green channel (Cy3) for mouse universal reference RNA (provided by the Genomics Core Facility). Hybridization was performed according to the standard protocol of “Two-Color Microarray-Based Gene Expression Analysis” for Agilent Gene Expression Oligo microarrays Version 5.0.1. Briefly, a 2× target mix was generated containing 125 ng cyanine 3- labeled cRNA, 125 ng cyanine 5-labeled cRNA, appropriate amounts of labeled synthetic target, and 25 µl of Agilent's 10× control solution in a final volume of 125 µl. The sample was then fragmented by the addition of 5 µl 25× fragmentation buffer followed by incubation at 60°C for 30 minutes. Samples were moved to ice, and fragmentation was stopped by addition of 125 µl of Agilent's 2× in situ hybridization buffer. Microarrays were hybridized in Agilent Microarray Hybridization Chambers for 17 hours at 60°C with mixing on an Agilent Rotator in a Robbin's Scientific Hybridization Oven. After hybridization, the arrays were scanned by an Axon GenePix 4000B scanner (Axon Instruments; Foster City, CA). The images were analyzed using Gene Pix Pro 5.0 software (Axon Instruments). Gene expression values were quantified by log base 2 ratio of red channel intensity (mean) and green channel intensity (mean), followed by Lowess normalization to remove the intensity-dependent dye bias. The raw data was submitted to NCBI's GEO database (Series GSE34278).

Data pre-processing was carried out via the UNC Microarray Database for quality filtering and data normalization. UNC Microarray Database (https://genome.unc.edu/) provides the service for microarray data storage, retrieval, analysis, and visualization to registered UNC-Chapel Hill researchers and their collaborators. Agilent array data was extracted on the probe level. For probes spotted multiple times, the mean expression value was computed and retained. All probe sequences were BLAT against the NCBI database [19] and were annotated with Entrez ID. When multiple probes were targeted on the same gene (with the same Entrez ID), these data were collapsed onto the Entrez ID, and mean values were computed as the gene expression value.

### Obtaining differentially expressed gene (DEG) and multivariate analyses

Pre-processed data were used to construct a series of data matrix files for further analysis. For a given data matrix, the rows were excluded if more than 40% of missing values were observed. The rest of missing data was imputed with a K-nearest neighbor (k = 9) approach. DEGs were obtained from two-class and multi-class statistical modeling using SAM (R package samr v.1.25) [20]. DEGs were obtained based on the corrected p-value≤0.05. When SAM was performed with Excel, DEGs were generated with the median number of false positives less than 1. To perform hierarchical clustering analysis [1], [21], [22], a data matrix with DEGs only was extracted, row median-centered and column-standardized. Clustering analysis was also performed with R (2.10.0). A separate principal component analysis (PCA) was further performed on each DEG dataset using the R bio3d package. PCA plots on the first three components were reported, and a scree plot was reported showing the accumulated variability explained by the first three principal components.

### Extraction of gene expression patterns

In order to show overall trends of the gene expression profile across the metaplasia phase, a pattern extraction method, the EPIG process, was applied [23]. Preprocessed and normalized data matrix and experimental design files were loaded into ExP software [24], the expression profile of each gene was compared exhaustively against all other genes, and statistically significant “profile patterns” were self-extracted and stored. Then the genes whose expression profiles supported the “profile patterns” were retained in their corresponding profile pattern gene lists and reported. Expression data matrices of the significant gene expression patterns obtained from EPIG were loaded into GeneSpring (Agilent Technologies) for pattern visualization.

### Gene set analysis (GSA)

GSA was carried out using R (GSA package). Curated gene sets in three major categories - canonical pathway (CP; 880 gene sets), transcription factor targets (TF; 615 gene sets), and Gene Ontology (GO; C5, 1,454 gene sets) - were downloaded from the GSEA web portal and used in this study (http://www.broadinstitute.org/gsea/index.jsp). Both two-class unpaired and multi-class comparisons were implemented based on the experimental design. 100 permutations were applied to generate a null distribution for statistical testing, and significantly enriched gene sets were obtained at a false discovery rate cutoff of 0.05–0.5. To ensure the validity of the analysis, in addition to the recommended GSA analysis, each analysis was repeated 100 times and the gene sets that showed in <10% of the repetitive studies were excluded from the final report. When GSA was performed in Excel, significantly enriched gene sets were obtained with a false discovery rate ≤0.5.

In addition, Fisher's exact test was performed against ten knowledge-based gene sets. These knowledge-based gene sets were manually collected from the literature. These genes are related to the structure of keratinized stratified squamous epithelium (i.e., basal lamina, basal layer, granular layer, spinous layer and keratinized layer), the epidermal differentiation complex (EDC), P63 target genes, Sox2 target genes, Pax9 target genes, and Nrf2/Keap1 target genes (Excel S1). P-values were reported based on the hypergeometric distribution, and gene sets with p-value≤0.05 were reported as significantly enriched gene sets within the DEG list.

### Analysis of archival data from the public database

Differential gene expression between E8.25 definitive endoderm and E11.5 esophagus has been studied previously using an Illumina Ambion microarray [8]. Raw microarray data of this study were downloaded from the Gene Expression Omnibus (GSE13040) under the accession numbers GSM326633–35 (E8.25) and GSM326642–44 (E11.5). Only probes which were significantly different from the background were used (p-value<0.05). To generate ratio data, the intensity of each probe on a single array was divided by the average intensity of the same probe on the rest of the arrays. Entrez ID was also used in Illumina Ambion microarray data. For probes without Entrez ID, GenBank accession numbers were used and then converted to Entrez ID.

### Real-time PCR

cDNA was prepared from DNase-treated total RNA using the Advantage RT-for-PCR Kit (Clontech; Mountain View, CA). TaqMan® Gene Expression Assays (FAM™ dye-labeled) with pre-designed primers for each target gene were obtained from Applied Biosystems (Foster City, CA). The six target genes were: *Pax9* (paired box gene 9, Assay ID: Mm00440629_m1); *Calm4* (calmodulin 4, Assay ID: Mm00490975_s1); *Sbsn* (suprabasin; Assay ID: Mm00552057_m1); *Ppard* (peroxisome proliferator activator receptor delta, Assay ID: Mm00803184_m1); *Pten* (phosphatase and tensin homolog, Assay ID: Mm00477208_m1); *Akt2* (thymoma viral proto-oncogene 2: Mm02026778_g1). *18S* (18S ribosomal RNA;hypothetical LOC790964, Assay ID: Mm03928990_g1) was used as the endogenous control. Relative quantitative real-time PCR was performed using an ABI 7900HT Fast Real-Time PCR System (Applied Biosystems) with SDS v2.3 software. The real-time data exported from RQ Manager 1.2 were further analyzed by DataAssist 3.0 (Applied Biosystems) to generate the RQ Plot.

### Histochemical and immunohistochemical staining

Tissues were routinely processed for paraffin sectioning (5 µm). H&E staining was carried out using a standard protocol. For X-Gal staining, mouse esophagi were isolated and fixed in 4% paraformaldehyde at 4°C for 20 min on ice. Staining and subsequent sample processing were performed as previously described [4], [6].

For immunohistochemical staining, the deparaffinized sections were submerged in methanol containing 0.3% hydrogen peroxide for 15 min at RT to inhibit endogenous peroxidase activity. Antigen retrieval was done prior to incubation with rabbit polyclonal anti-Nrf2 (#PA1-38312, 1∶40; Thermo Scientific, Waltham, MA), or rabbit polyclonal anti-Pparβ/δ (#LS-B45, 1∶1,000; LifeSpan Biosciences, Seattle, WA), or rabbit polyclonal anti-pAkt(Ser473) (#3787, 1∶25; Cell Signaling Technology, Danvers, MA), overnight at 4°C. Tissue sections were then washed again in PBS and incubated with peroxidase-conjugated secondary antibodies for 30 minutes at 37°C. Detection of the antibody complex was done using the streptavidin-peroxidase reaction kit with DAB as a chromogen (ABC kit; Vector Labs, Burlingame, CA). To ensure the specificity of the primary antibody, control tissue sections were incubated in the absence of primary antibodies.

## Results

In this study, we divided the developmental process of mouse esophageal epithelium into three phases based on morphological changes (Figure 1A): (a) The specification phase is defined as the phase during which the definitive endoderm differentiates into the esophagus. Two time points, E8.25 and E11.5, were chosen to represent this phase. (b) The metaplasia phase is defined as the phase during which the simple columnar epithelium in the esophagus undergoes metaplastic changes (stratification, squamation and keratinization) into a keratinized stratified squamous epithelium. Four time points, E11.5, E15.5, P0 and P7, were selected to represent this phase. (c) The maturation phase is defined as the phase during which the keratinized stratified squamous epithelium continues to thicken and finally forms the esophageal epithelium in adults. Two time points, P7 and adult, were selected to represent this phase. In the metaplasia phase, the esophagus is covered by a simple columnar epithelium surrounded by a well-defined but undifferentiated mesenchyme at E11.5 (Figure 1B, F). At E15.5, it becomes stratified, consisting of ∼3 cell layers, with well-defined submucosa and muscle (Figure 1C, G). At P0, epithelial cells lose columnar features and appear squamous. The esophagus is covered by a stratified squamous epithelium with 3–5 cell layers surrounded by a mesenchyme consisting of thicker muscle (Figure 1D, H). At P7, a keratinized layer has clearly formed at the surface of the epithelium, and the base membrane and submucosal papillae are well-formed (Figure 1E, I).

### 1. Gene expression profiles during the development of wild-type mouse esophagus

#### a. Specification phase

Two-class SAM analysis identified 1,612 genes up-regulated and 1,303 genes down-regulated in E11.5 esophagi as compared with E8.25 definitive endoderm (Excel S2). Hierarchical clustering analysis and PCA analysis showed that E8.25 definitive endoderm and E11.5 esophagus were clustered separately (Figure S1). Among the up-regulated genes, *Irf6, Sox21, Nfib, Upk2, Hoxa5, Sox2, P63, Foxq1, Hoxa2, Hoxa4, Ovol2, Emp1, Lhfp, Kremen2, Twist1, Rarb, Hoxb4, Nfe2l3, Erf* and *Hoxc6* were reported exclusively or highly expressed in E11.5 esophagus as compared to other definitive endoderm-derived organs [8]. Furthermore, several signaling pathway-related genes, such as *Klf5* (TGFβ signaling), *Shh* and *FoxA2* (Hedgehog signaling), and *β-catenin* (Wnt signaling), were up-regulated in E11.5 esophagus as compared with E8.25 definitive endoderm, suggesting these pathways were likely involved in esophageal specification.

GSA analysis identified multiple enriched gene sets in the categories of canonical pathway, gene ontology and transcription factor (Excel S2). For example, GO_629 (morphogenesis of an epithelium), GO_727 (epidermis development), and GO_1049 (ectoderm development) were enriched in E11.5 esophagus. However, using Fisher's exact test, only *P63* target genes were significantly different between E8.25 definitive endoderm and E11.5 esophagus (Excel S2).

#### b. Metaplasia phase

Multi-class SAM analysis identified 2,076 DEGs at this phase (Excel S3). Hierarchical clustering and PCA analysis clearly showed that three samples at each time point were clustered together (Figure S2). As expected, E11.5 and E15.5 were separated from P0 and P7. Among the 2,076 DEGs, many genes are known to be involved in differentiation and function of keratinocytes; these processes include keratinization (*Cnfn, Ctnnd, Evpl, Fgf10, Krt10, Krt17, Krt36, Krt79, Krt80, Krt84, Ppl, Ptch1, Tgfb2*), gap junction (*Csda, Gjb2, Gjb3, Gjb4 Gjb6, Gjd4*), muscle development (*Mybpc2, Mybph, Myh1, Myh10, Myh2, Myl1, Myl3, Myo18b, Myo5b, Myo6, Myom1, Bmp4*), blood vessel development (*Tgfbr3, Tgm2, Fgf10, Col3a1, Edn1, Edn2, Epas1, Agt*), and neuron development (*Bdnf, Cacng4, Dlx2, Dmd, Epha7, Erbb3, Hoxb3, Nrtn*).

Eighteen gene expression patterns were extracted from DEGs (Excel S4). Pattern 1 showed 763 genes up-regulated from E11.5 to P0, and Pattern 2 showed 369 genes down-regulated from E11.5 to P0. These genes were associated with metaplasia. Pattern 4 (188 genes), Pattern 11 (65 genes), Pattern 13 (33 genes), Pattern 15 (13 genes), and Pattern 16 (7 genes) were up-regulated from E11.5 to E15.5 and stayed at the same level or were down-regulated after E15.5. Genes in Pattern 7 (5 genes), Pattern 12 (18 genes), and Pattern 17 (5 genes) were down-regulated from E11.5 to E15.5 and stayed at the same level afterwards. These genes were probably involved in stratification of columnar epithelial cells. From E15.5 to P0, Pattern 3 (180 genes), Pattern 8 (40 genes), Pattern 10 (37 genes) and Pattern 15 were up-regulated, and Pattern 5 (79 genes), Pattern 11, Pattern 13 and Pattern 16 (7 genes) were down-regulated. These genes were probably involved in squamation. From P0 to P7, Pattern 6 (51 genes), Pattern 12 (18 genes), Pattern 14 (16 genes), and Pattern 18 (5 genes) were up-regulated, and Pattern 9 (18 genes), Pattern 10, Pattern 11, and Pattern 15 were down-regulated. These genes were probably involved in keratinization, as supported by the fact that genes in Pattern 1, Pattern 3, Pattern 4, Pattern 6 were generally up-regulated from E11.5 to P7. As expected, these genes (*Muc4, Ppl, Arg1, Ocln, Bmp6, Tchh, Trp73, Lces, Krts, Sprrs*) were known to be associated with keratinized stratified squamous epithelium.

We collated ten knowledge-based gene sets from the literature (Excel S1). These gene sets are associated with differentiation of the skin, the esophagus and the tongue, all of which are covered by keratinized stratified squamous epithelia. Fisher's exact test of our data showed that nine gene sets were significantly associated with esophageal development in the metaplasia phase: basal lamina genes, basal layer genes, granular layer genes, keratinized layer genes, EDC genes, *P63* target genes, *Pax9* target genes, *Sox2* target genes and Nrf2/Keap1 pathway genes (Table 1). The expression patterns of these gene sets throughout the metaplasia phase were generated by GeneSpring to demonstrate dynamic changes (Excel S4). It is clear that genes of EDC and epithelial layers were generally up-regulated throughout this phase. This is consistent with the morphological change of esophageal epithelium: the transition from simple columnar epithelium to keratinized stratified squamous epithelium. *Pax9* target genes were also generally up-regulated during the metaplasia phase, suggesting a critical role of *Pax9* in esophageal epithelial differentiation. Real-time PCR confirmed increasing expression of *Pax9* and its downstream keratinization-associated genes (*Calm4* and *Sbsn*) from E15.5 to P7 (Figure S3).

**Table 1:** Differential expression of knowledge-based gene sets in the mouse esophagus in the metaplasia phase.

Samples	Knowledge-based gene set	No. of genes in the gene set	No. of genes in array dataset	No. of DEGs	P value
Wild-type E11.5 vs E15.5 vs P0 vs P7	Basal lamina genes	40	29	2	0.222
	Basal layer genes	14	10	1	0.267
	Spinous layer genes	11	7	0	1.000
	Granular layer genes	16	10	1	0.267
	Keratinized layer genes	39	14	4	6.9E-4
	EDC genes	58	21	3	0.029
	Nrf2/Keap1 pathway genes	281	181	31	1.8E-14
	P63 target genes	59	39	4	0.031
	Pax9 target genes	23	13	3	0.007
	Sox2 target genes	141	80	6	0.036
Wild-type vs *Keap1* ^−/−^ at P7	Basal lamina genes	40	29	16	3.0E-7
	Basal layer genes	14	10	4	0.041
	Granular layer genes	16	10	4	0.043
	Spinous layer genes	11	7	1	0.661
	Keratinized layer genes	39	14	9	2.6E-5
	EDC genes	58	21	9	0.001
	Nrf2/Keap1 pathway genes	281	181	37	0.019
	P63 target genes	59	39	21	8.9E-9
	Pax9 target genes	23	11	9	1.1E-6
	Sox2 target genes	141	80	20	0.008

In order to explore potential involvement of biochemical pathways, signaling pathways and transcription factors, multi-class and two-class GSA analyses were performed (Excel S3). It is clear that from E11.5 to E15.5, the epithelial structure gene sets (GO_55, GO_60, GO_66, GO_727), glutathione transfer gene set (*Nrf2*-relevant, GO_1418), and Ppar signaling pathway (CP_80) were up-regulated, while TGFβ signaling pathways (CP_110, CP_381, CP_699) were down-regulated. From E15.5 to P0, a keratinocyte gene set (CP_295) was up-regulated. The TLR and NFκB pathways (CP_122, CP_312, CP_392, CP_727, CP_728, CP_730, CP_838) were up-regulated from E15.5 to P0, and several of these were down-regulated from P0 to P7. The Wnt pathway (CP_851) and Hedgehog pathway (CP_170, CP_109) were down-regulated in P7 as compared with E11.5. These data suggest that Wnt, NFκB, TGFβ, Hedgehog and Nrf2/Keap1 pathways are very likely involved in the metaplasia phase during the development of esophageal epithelium.

**Figure 2:**
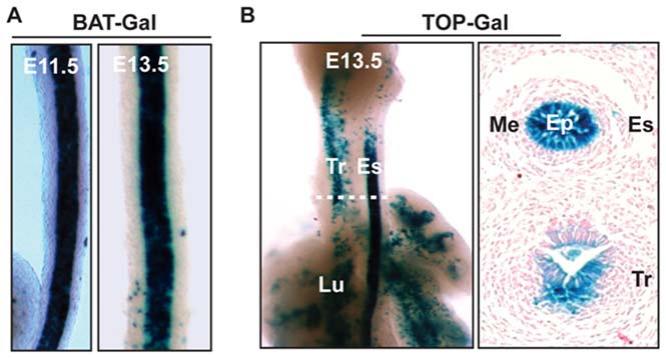
Involvement of the Wnt pathway in the development of mouse esophageal epithelium. (A) X-Gal staining of E11.5 and E13.5 esophagi of BAT-GAL mice; (B) X-Gal staining of E13.5 esophagi of TOP-GAL mice. Es, esophagus; Tr, trachea; Ep, epithelium; Me, mesenchyme; Lu, lung.

Using the Wnt pathway as an example, we examined its potential involvement in esophageal epithelial development using two mouse lines (BAT-lacZ and TOPGAL) that have been routinely used to report Wnt signaling [17], [18]. Consistent with the microarray data, both mouse lines indicated that Wnt signaling was active in the developing esophagus between E11.5–E13.5. Sections of X-gal stained sample showed that Wnt signaling was limited to the epithelium at E11.5. At E13.5 minimal activity was also noticed in the mesenchyme, whereas the epithelium remained strongly positive for X-gal staining (Figure 2). After E13.5, Wnt activity decreased in the epithelium, and activity disappeared at E17.5.

#### c. Maturation phase

**Figure 3:**
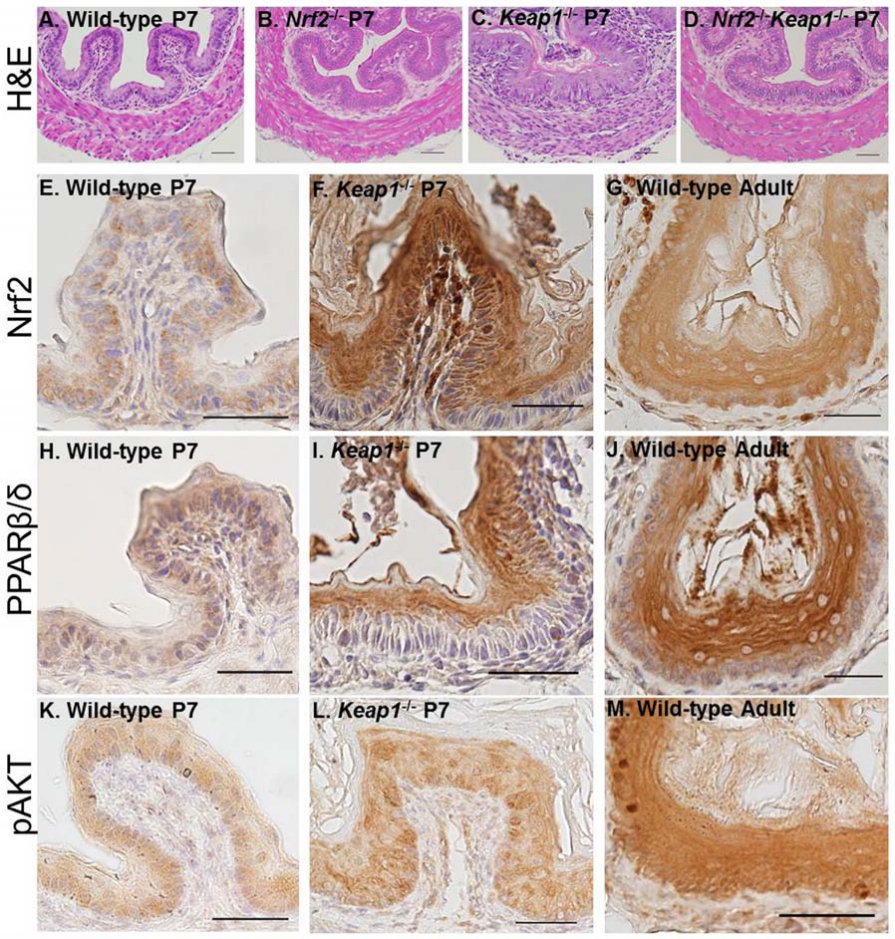
Esophageal hyperkeratosis due to *Nrf2* superactivation in *Keap1^−/−^* esophagus. P7 esophagi of a wild-type mouse (A), a *Nrf2* ^−/−^ mouse (B), a *Keap1* ^−/−^ mouse (C), and a *Nrf2* ^−/−^ *Keap1* ^−/−^ mouse (D), were stained for H&E. Expression of Nrf2 (E–G), Pparγ/δ (H–J) and pAkt (K–L) were shown in the esophagi of P7 wild-type mouse, P7 *Keap1* ^−/−^ mouse and adult wild-type mouse. Size bar = 50 µm.

Comparing wild-type P7 with adult, we found 1,248 genes up-regulated and 587 genes down-regulated in adult esophageal epithelium. Among the up-regulated genes, many were known *Nrf2* target genes, such as *Akr1b8, Aldhs, Mts, Hmox1, Gsts, Abccs, Nqo1, Ltb4dh* and *Nrf2* itself. GSA analysis shows that four *Nrf2*-relevant pathways (CP_29, CP_67, CP_68, CP_530) were up-regulated in adult esophagi as compared to P7 esophagi. Ppar signaling pathways (CP_80, CP_101, CP_623) were up-regulated, and Notch signaling (CP_108, CP_696) was down-regulated (Excel S5). Fisher's exact test showed that the Nrf2/Keap1 pathway, basal lamina genes, basal layer genes, granular layer genes, spinous layer genes and *P63* target genes were significantly enriched in the adult epithelium (Excel S5). These data suggest that in the maturation phase the Nrf2/Keap1 pathway is further activated in mouse esophageal epithelium above the baseline activity in the metaplasia phase. Consistent with these data, we found overexpression of Nrf2 in adult esophagi as compared to P7 esophagi (Figure 3E, G). Meanwhile, Pparβ/δ and pAkt expression was correlated with Nrf2 expression (Figure 3H, J; K, M). These data further supported the Pparβ/δ and PI3K/Akt pathway as possible *Nrf2* downstream effectors promoting keratinization.

### 2. The role of Nrf2/Keap1 pathway during the development of mouse esophagus

In specification phase, neither individual genes nor gene sets associated with Nrf2/Keap1 pathway was selected by SAM or GSA (Excel S2), suggesting that the Nrf2/Keap1 pathway was unlikely involved in the specification phase during mouse esophageal epithelial development.

#### a. Metaplasia phase

In the metaplasia phase, 37 Nrf2 target genes were selected as differentially expressed by multi-class SAM (Excel S3). Fisher's exact test with these genes showed that the Nrf2/Keap1 pathway was significantly associated with metaplasia phase (P = 0.019422) (Table 1).

In order to further examine the role of Nrf2/Keap1 pathway in the metaplasia phase, we profiled gene expression in P7 esophagi from wild-type, *Nrf2* ^−/−^, *Keap1* ^−/−^ and *Nrf2* ^−/−^ *Keap1* ^−/−^ mice. At this time point, both *Nrf2* ^−/−^ and *Nrf2* ^−/−^ *Keap1* ^−/−^ esophagi appeared normal, whereas *Keap1* ^−/−^ esophagus appeared hyperkeratotic (Figure 3A–D). Multi-class SAM analysis identified 526 DEGs (Excel S6). Two-class SAM confirmed that the major difference among these four groups was between the wild-type and *Keap1* ^−/−^ esophagi (Excel S6). In agreement with these data, hierarchical clustering analysis and PCA analysis clearly demonstrated separation of the *Keap1* ^−/−^ esophagi from others (Figure S4). Between wild-type and *Nrf2* ^−/−^ esophagi, only one gene (*Rbm45*) was up-regulated, and three genes including *Nrf2* were down-regulated in *Nrf2* ^−/−^ esophagi. Between wild-type and *Nrf2* ^−/−^ *Keap1* ^−/−^esophagi, 19 genes were up-regulated and 10 genes including *Nrf2*, *Upk3a* and *Krt17* were down-regulated in *Nrf2* ^−/−^ *Keap1* ^−/−^ esophagi. In constrast, 309 genes were up-regulated and 346 genes down-regulated in *Keap1* ^−/−^ esophagi as compared with wild-type esophagi. Among the up-regulated genes, many classical *Nrf2* target genes were enriched, such as *Nqo1, Gclm, Gclc, Gsts, Cat, Cyps, Mts, Mgsts, Aldhs, Cess and Abccs*, indicating *Nrf2* superactivity. Keratinization-related genes such as *Sprr2h, Krt84, Ptgs2, Casp14* and *Ppard* were also up-regulated in *Keap1* ^−/−^ esophagi. These data suggest that although the Nrf2/Keap1 pathway was involved in the metaplasia phase as shown above, *Nrf2^−/−^* did not have any significant impact on gene expression in the esophagus. This observation may be explained by compensation by other genes or a low baseline activity in this phase. However, hyperactive *Nrf2* due to *Keap1* ^−/−^ activated the Nrf2/Keap1 pathway in the esophagus, and hence up-regulated downstream target genes.

Among the 10 knowledge-based gene sets, Fisher's exact test identified six gene sets significantly different in *Keap1* ^−/−^ esophagi as compared to wild-type esophagi: keratinized layer genes, EDC genes, *P63* target genes, *Pax9* target genes, *Sox2* target genes and Nrf2/Keap1 pathway genes (Table 1). As expected, the Nrf2/Keap1 pathway genes were highly significant (p = 1.8×10^−14^). The keratinized layer genes, EDC genes and *Pax9* target genes were known to be associated with keratinization of stratified squamous epithelium. These data were consistent with the phenotype of esophageal hyperkeratosis in *Keap1* ^−/−^ mice.

An interesting question is why *Keap1* ^−/−^ mice developed esophageal hyperkeratosis. Two-class GSA analysis was performed to identify gene sets associated with the *Keap1* ^−/−^ esophagi as compared with wild-type esophagi. Among the enriched gene sets (Excel S6), the Nrf2/Keap1 transcription factors were significantly up-regulated in *Keap1* ^−/−^ esophagi (*Nfe2, Nrf2, Srebp1*), as well as *Nrf2*-relevant metabolism GO gene sets (GO_666, GO_1221, GO_1333, GO_1374, GO_1408, GO_1418) and canonical pathway gene sets (CP_29, CP_67, CP_68, CP_71, CP_429, CP_530, CP_625, CP_626). In addition to these, Ppar pathway (CP_80 and CP_101) and PI3K/Akt pathway (CP_629 and CP_435) were also up-regulated in *Keap1* ^−/−^ esophagi, suggesting potential roles of these pathways in superactive *Nrf2*-induced esophageal hyperkeratosis in *Keap1* ^−/−^ mice. Real-time PCR showed that *Ppard* was up-regulated and *Pten* down-regulated in *Keap1* ^−/−^ esophagus, while keratinization-associated genes (*Calm4* and *Sbsn*) were up-regulated (Figure S6).

Among three Ppar isoforms, Pparβ/δ activation is known to cause terminal differentiation of keratinocytes [25], and *Pparβ/δ* was up-regulated in *Keap1* ^−/−^ esophagi as compared with wild-type esophagi (Excel S6). Keratinocyte-specific deficiency of *Pten* caused Akt activation, and subsequently resulted in postnatal death due to esophageal hyperkeratosis [26]. Here we examined expression of Nrf2, Pparβ/δ and pAkt in the P7 esophageal epithelium of wild-type and *Keap1* ^−/−^ mice. Consistent with the expression pattern reported in the literature [27], *Nrf2* was found to translocate into the nuclei of esophageal epithelial cells in *Keap1* ^−/−^ mice (Figure 3E, F). Corresponding to Nrf2 activation, Pparβ/δ and pAkt were also overexpressed in the cytoplasm and nuclei (Figure 3H, I; K, L). These data suggested that hyperactive Nrf2 might promote esophageal hyperkeratosis in *Keap1* ^−/−^ mice through activation of the Pparβ/δ and PI3K/Akt pathway.

#### b. Maturation phase

Further analysis of adult wild-type and *Nrf2* ^−/−^ esophagi showed that 11 genes were up-regulated and 25 down-regulated (including Nrf2 and its target genes), in *Nrf2* ^−/−^ esophagi (Excel S7). Among these 25 genes, *Akr1b8, Nqo1, Gstm3, Nrf2, Gsta3, Gstm1* and *Gclc* are known as classical *Nrf2* target genes. Hierarchical clustering and PCA analysis clustered wild-type and Nrf2^−/−^ esophagi separately (Figure S5). Based on three lines of evidence, we concluded that *Nrf2* was mainly involved in maturation phase from P7 to adulthood: (1) there was little difference in gene expression between wild-type and *Nrf2* ^−/−^ esophagus at P7; (2) Nrf2/Keap1 pathway genes were differentially expressed between P7 and adult esophagus of wild-type mice; (3) Nrf2/Keap1 pathway genes were differentially expressed between adult wild-type and *Nrf2* ^−/−^ mice. These genes are known to function in detoxification and anti-oxidative defense.

## Discussion

This study clearly demonstrated a complex mechanism involving many genes and pathways at each phase during the development of mouse esophageal epithelium. There was a baseline activity of the Nrf2/Keap1 pathway in the metaplasia phase, and a higher activity in the maturation phase. Hyperactive *Nrf2* in *Keap1* ^−/−^ mice resulted in esophageal hyperkeratosis, probably through activation of the Pparβ/δ and PI3K/Akt pathway.

Our data were consistent with previous studies on mouse esophageal development. *P63* and *Sox2* were expressed in the mouse esophagus prior to E11.5, suggesting their critical roles in esophageal specification [6]. *Pax9* was expressed in the mouse esophagus at E13.5 [28] and was essential for expression of multiple genes in the keratinized layer or EDC of mouse tongue [29]. The Wnt pathway promoted respiratory progenitor identity in the mouse foregut, and continuous activation of the Wnt pathway resulted in the reprogramming of esophagus and stomach to a lung endoderm progenitor fate [30], [31]. This explains why the Wnt pathway became inactive in the esophagus later in the metaplasia phase (Figure 2). The NFκB pathway, especially *IKKα*, played an important role in keratinocyte differentiation [32]. Hedgehog pathway participated in esophageal development by signaling from the endoderm to the mesoderm [33], [34]. Bmp pathway was inhibited between E10.5 and E14.5 to allow metaplasia to take place. After E14.5–E15.5, active Bmp signaling is required for further differentiation of esophageal epithelium [4].

Our main goal in this study was to determine the role of the Nrf2/Keap1 pathway in the development of esophageal epithelium. Using gene microarray analysis with wild-type mouse samples, we found that the Nrf2/Keap1 pathway was likely uninvolved in the specification phase (Excel S2). Starting from the metaplasia phase, there was a baseline activity of the Nrf2/Keap1 pathway. However, *Nrf2* ^−/−^ did not have a significant impact on gene expression and morphology of esophageal epithelium at P7 (Excel S6). We believe that the Nrf2/Keap1 pathway is mainly involved in the development of esophageal epithelium in the maturation phase (Excel S5). As compared with wild-type adult mice, *Nrf2* ^−/−^ reduced expression of multiple downstream genes whose major functions are detoxification and anti-oxidative defense (Excel S7).

It is unknown why hyperactive Nrf2 in Keap1^−/−^ mice caused hyperkeratosis of the esophageal epithelium at P7. Similar to the esophagus, the skin was also hyperkeratic in *Keap1* ^−/−^ mice [15], suggesting similar mechanisms of hyperkeratosis in the skin and the esophagus. Mechanistically, *Nrf2* is known to regulate *Krt16*/*Krt17* expression through MAP kinases [35]. In this study, GSA analysis identified two potential candidate pathways responsible for hyperkeratosis: Ppar signaling and PI3K/Akt pathway (Excel S6). Although *Pparγ* is a direct transcriptional target of *Nrf2* [36], *Pparβ/δ* is more likely to be the isoform involved among the three Ppar isoforms because Pparβ/δ agonists were known to cause terminal differentiation of keratinocytes *in vitro* [25], [37] and dermal hyperkeratosis *in vivo* [38]. While *Pparβ/δ* ^−/−^ inhibited epidermal keratinization, transgenic overexpression promoted epidermal hyperkeratosis [39], [40]. Several *Nrf2* target genes (*Aldh3a1*, *Gstm3*, *Gsto1*, *Gsta1*, *Aldh9a1*) were also known to be regulated by *Pparβ/δ* [41]. In this study, we confirmed overexpression of *Pparβ/δ* in *Keap1* ^−/−^ esophagus relative to wild-type esophagus at P7. Adult esophagus also expressed a higher level of *Pparβ/δ* than P7 esophagus, which is less keratinized (Figure 3 H, I, J). These data supported the hypothesis that *Keap1* ^−/−^ might produce esophageal hyperkeratosis through activation of *Pparβ/δ.*

Other than *Pparβ/δ*, the PI3K/Akt pathway may also contribute to hyperactive Nrf2-induced esophageal hyperkeratosis (Figure 3 K, L, M). Keratinocyte-specific deficiency of *Pten* caused Akt activation, and subsequently resulted in postnatal death due to esophageal hyperkeratosis [26]. Notch pathway is the third candidate. Recent studies demonstrated regulation of the Notch pathway by Nrf2 [42] and participation of the Notch pathway in terminal differentiation of esophageal epithelium [43]. Further studies are warranted to identify downstream effectors that contribute to esophageal hyperkeratosis.

Esophageal hyperkeratosis in humans may develop as a result of vitamin A deficiency, vitamin E excess, HPV-induced papillomatosis, Darier's disease, tylosis or caustic injury [44]. It is also commonly seen in rodent models of esophageal cancer or reflux esophagitis. We suspect that the Nrf2/Keap1 pathway is involved in some of these cases. For example, retinoic acid is known to inhibit Nrf2 [45]. Vitamin A deficiency may cause Nrf2 hyperactivity and esophageal hyperkeratosis. In addition to a mechanistic understanding of human esophageal disease, manipulation of the Nrf2/Keap1 pathway may provide a novel way of enhancing the protective barrier of the esophageal epithelium. The keratinized layer is the major protective layer against physical stress and chemical injuries [46]. Terminally differentiated keratinocytes express proteins which can provide protection by quenching reactive oxygen species [47]. In fact, sulforaphane, a chemical activator of Nrf2, restores skin integrity in an epidermolysis bullosa simplex model (created by *Krt5* or *Krt14* mutation) by activating *Krt17* expression [48]. Similarly, Pparβ/δ activation can also enhance the epidermal permeability barrier [25], [38].

This study has many potential implications for future studies. Several developmental pathways involved in esophageal development were found to be active at an early time point and then became inactive later on (Table 2). However, these pathways are known to be involved in esophageal diseases such as Barrett's esophagus and esophageal cancer, suggesting that tight spatiotemporal regulation of these pathways is critical for both development and disease [49], [50], [51], [52], [53], [54]. Further understanding of these pathways during development will shed light on molecular mechanisms of esophageal diseases.

**Table 2:** Pathway changes in the three phases of mouse esophageal development.

Signaling pathway	Developmental phase		
	Specification	Metaplasia	Maturation
Wnt	↑	↑↓	
Hedgehog	↑	↓	
TGFβ	↑	↓	
BMP^a^		↓↑	
NFκB		↑↓	
Notch^b^			↑
Nrf2/Keap1			↑

Note: ↑ and ↑ indicate up- or down-regulation, respectively.

a Reference [4].

b Reference [43].

## Supporting Information

Figure S1 Hierarchical clustering analysis and PCA analysis of gene expression array data of wild-type mouse definitive endoderm (E8.25) and esophagi (E11.5): (A) clustering analysis; (B) PCA analysis.
(TIF)

Figure S2 Hierarchical clustering analysis and PCA analysis of gene expression array data of wild-type mouse esophagi (E11.5, E15.5, P0, P7): (A) clustering analysis; (B) PCA analysis.
(TIF)

Figure S3 Real-time PCR analysis of mRNA expression in wild-type mouse esophagi: relative mRNA levels of *Pax9* and its target genes (*Sbsn*, *Calm4*) in mouse esophageal epithelium of E15.5, P0, P7 and adult mice.
(TIF)

Figure S4 Hierarchical clustering analysis and PCA analysis of gene expression array data of P7 mouse esophagi (wild-type, *Nrf2* ^−/−^, *Keap1* ^−/−^, *Nrf2* ^−/−^ *Keap1* ^−/−^): (A) clustering analysis; (B) PCA analysis.
(TIF)

Figure S5 Hierarchical clustering analysis and PCA analysis of gene expression array data of mouse esophagi (wild-type adult, *Nrf2* ^−/−^ adult): (A) clustering analysis; (B) PCA analysis.
(TIF)

Figure S6 Real-time PCR analysis of mRNA expression in wild-type and *Keap1* ^−/−^ mouse esophagi: relative mRNA levels of *Pax9*, *Sbsn*, *Calm4*, *Ppard*, *Pten* and *Akt2* in the whole esophagi of wild type and *Keap1* ^−/−^ mice at P7.
(TIF)

Excel S1 Knowledge-based gene sets and references.
(XLS)

Excel S2 Differential gene expression in the specification phase during wild-type mouse esophageal development: (1) Raw gene expression array data after data pre-processing (E8.25 endoderm and E11.5 esophagus); (2) SAM analysis of differentially expressed genes (E8.25 endoderm vs E11.5 esophagus); (3) GSA analysis of differentially expressed gene sets (E8.25 endoderm vs E11.5 esophagus); (4) Fisher's exact test of knowledge-based gene sets (E8.25 endoderm vs E11.5 esophagus).
(XLSX)

Excel S3 Differential gene expression in the metaplasia phase during wild-type mouse esophageal development: (1) Raw gene expression array data after data pre-processing (E11.5, E15.5, P0 and P7); (2) SAM analysis of differentially expressed genes (E11.5 vs E15.5 vs P0 vs P7); (3) GSA analysis of differentially expressed gene sets (E11.5 vs E15.5 vs P0 vs P7).
(XLSX)

Excel S4 Dynamic expression of eighteen gene expression patterns and nine knowledge-based gene sets in the metaplasia phase.
(XLSX)

Excel S5 Differential gene expression in the maturation phase during the development of wild-type mouse esophageal epithelium: (1) Raw gene expression array data after data pre-processing (P7 and adult esophagus); (2) SAM analysis of differentially expressed genes (P7 vs adult esophagus); (3) GSA analysis of differentially expressed gene sets (P7 vs adult esophagus); (4) Fisher's exact test of knowledge-based gene sets (P7 vs adult esophagus).
(XLSX)

Excel S6 Differential gene expression at P7 between wild-type mice, *Nrf2* ^−/−^ mice, *Keap1* ^−/−^ mice, and *Nrf2* ^−/−^ *Keap1* ^−/−^ mice: (1) Raw gene expression array data after data pre-processing (wild-type P7, *Nrf2* ^−/−^ P7, *Keap1* ^−/−^ P7, *Nrf2* ^−/−^ *Keap1* ^−/−^ P7); (2) SAM analysis of differentially expressed genes (wild-type P7 vs *Nrf2* ^−/−^ P7 vs *Keap1* ^−/−^ P7 vs *Nrf2* ^−/−^ *Keap1* ^−/−^ P7); (3) GSA analysis of differentially expressed gene sets (wild-type P7 vs *Keap1* ^−/−^ P7).
(XLSX)

Excel S7 Differential gene expression between esophageal epithelium of adult wild-type and *Nrf2* ^−/−^ mice: (1) Raw gene expression array data after data pre-processing (wild-type adult, *Nrf2* ^−/−^ adult); (2) SAM analysis of differentially expressed genes (wild-type adult vs *Nrf2* ^−/−^ adult).
(XLSX)
