# Correction: Transcript Profiling Identifies Dynamic Gene Expression Patterns and an Important Role for Nrf2/Keap1 Pathway in the Developing Mouse Esophagus

**DOI:** 10.1371/annotation/b2554ac2-5eec-4fc1-b625-c79f0cc064ed

**Published:** 2012-05-23

**Authors:** Hao Chen, Jianying Li, Haiyan Li, Yuhui Hu, Whitney Tevebaugh, Masayuki Yamamoto, Jianwen Que, Xiaoxin Chen

Funding should read "Takeda MA-NC-D-156, NIH U54 CA156735 and NIH DK082650. The funders had no role in study design, data collection and analysis, decision to publish, or preparation of the manuscript." 

