# Supplementary figures and images for: Transcript Profiling Identifies Dynamic Gene Expression Patterns and an Important Role for Nrf2/Keap1 Pathway in the Developing Mouse Esophagus
Source: PLoS One. 2012 May 2;7(5):e36504. doi: 10.1371/journal.pone.0036504 (PMC3342176; doi:10.1371/journal.pone.0036504)

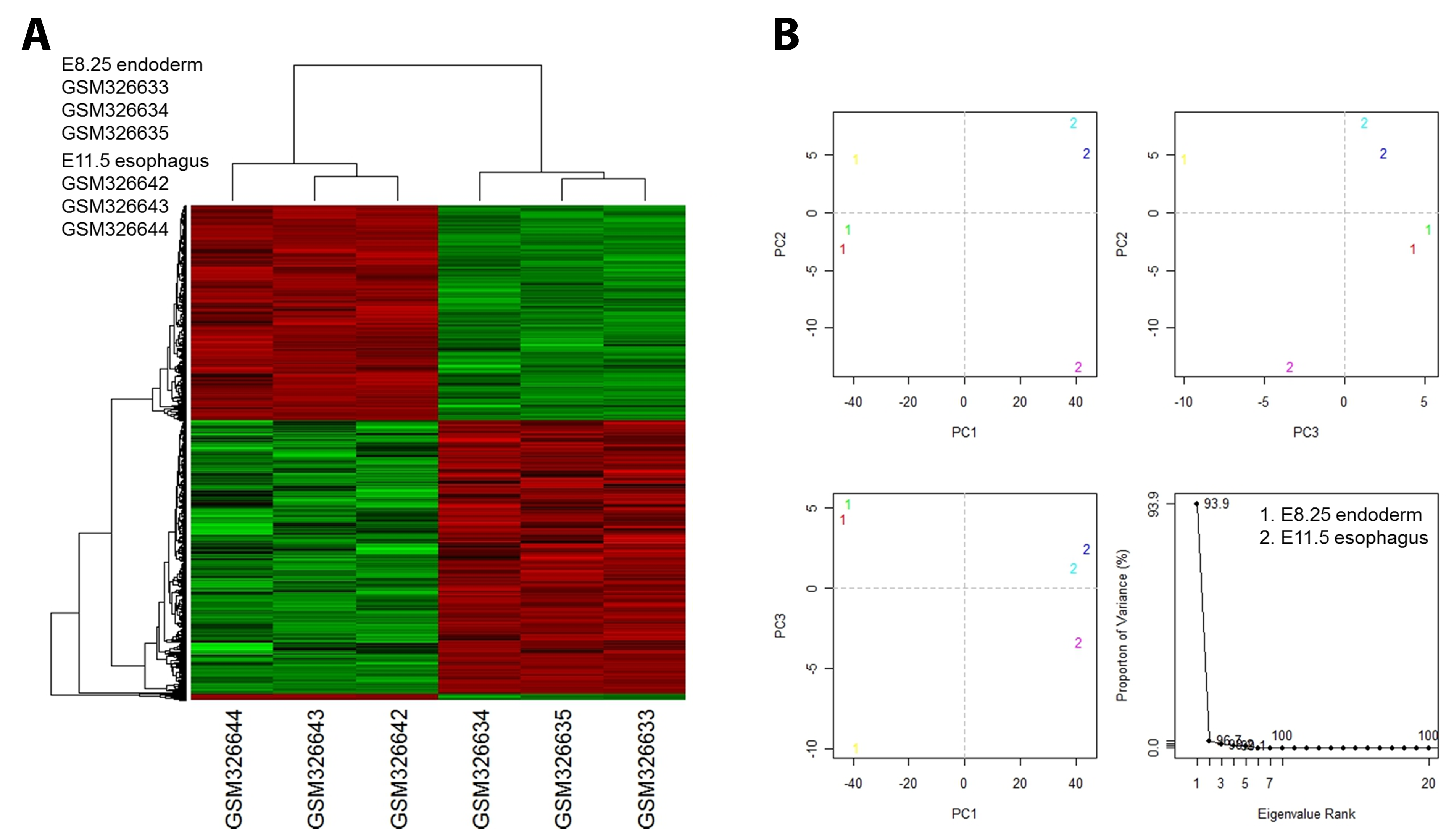

Supplement: Figure S1 — Hierarchical clustering analysis and PCA analysis of gene expression array data of wild-type mouse definitive endoderm (E8.25) and esophagi (E11.5): (A) clustering analysis; (B) PCA analysis. (TIF) [file pone.0036504.s001.tif]

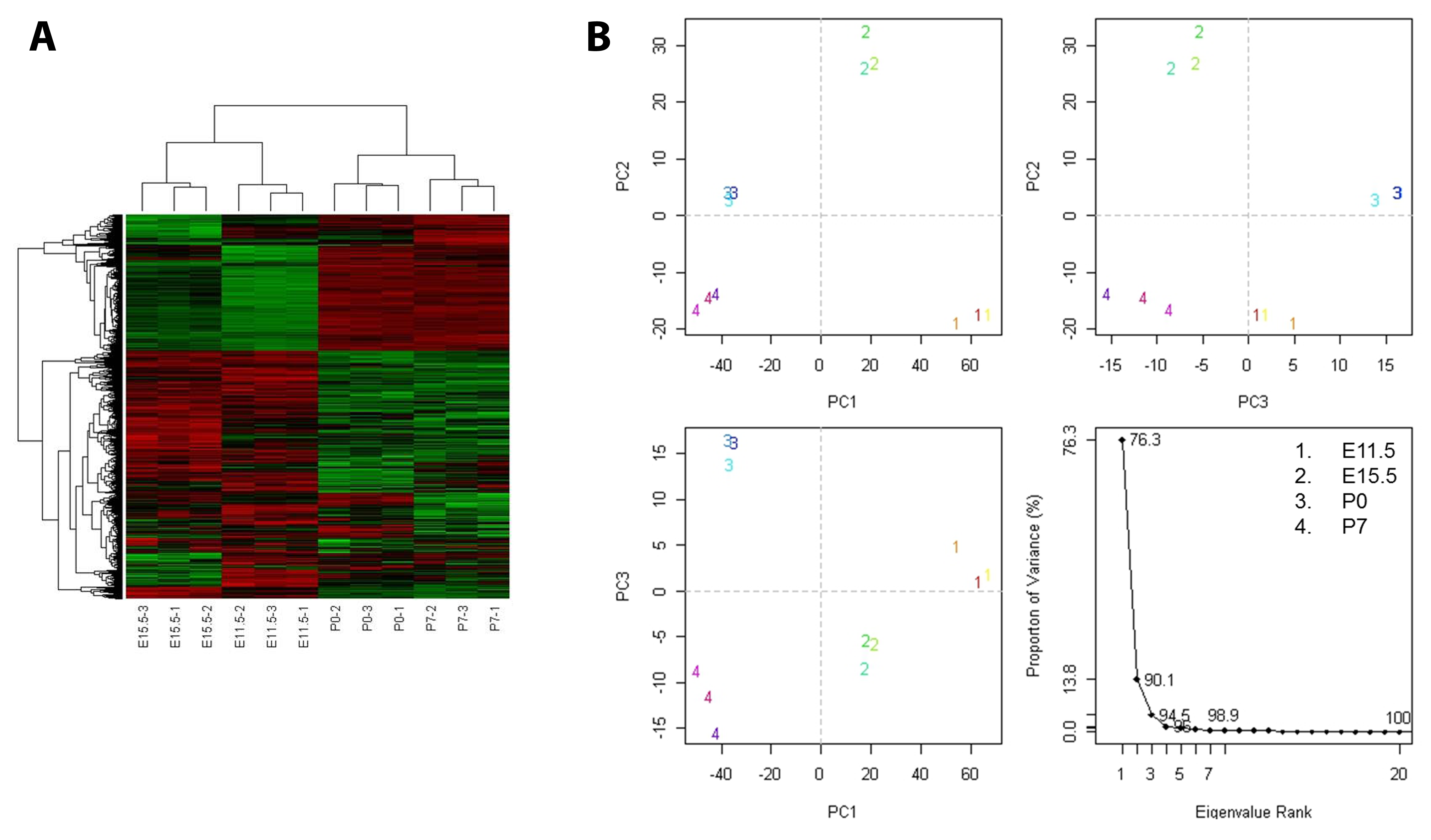

Supplement: Figure S2 — Hierarchical clustering analysis and PCA analysis of gene expression array data of wild-type mouse esophagi (E11.5, E15.5, P0, P7): (A) clustering analysis; (B) PCA analysis. (TIF) [file pone.0036504.s002.tif]

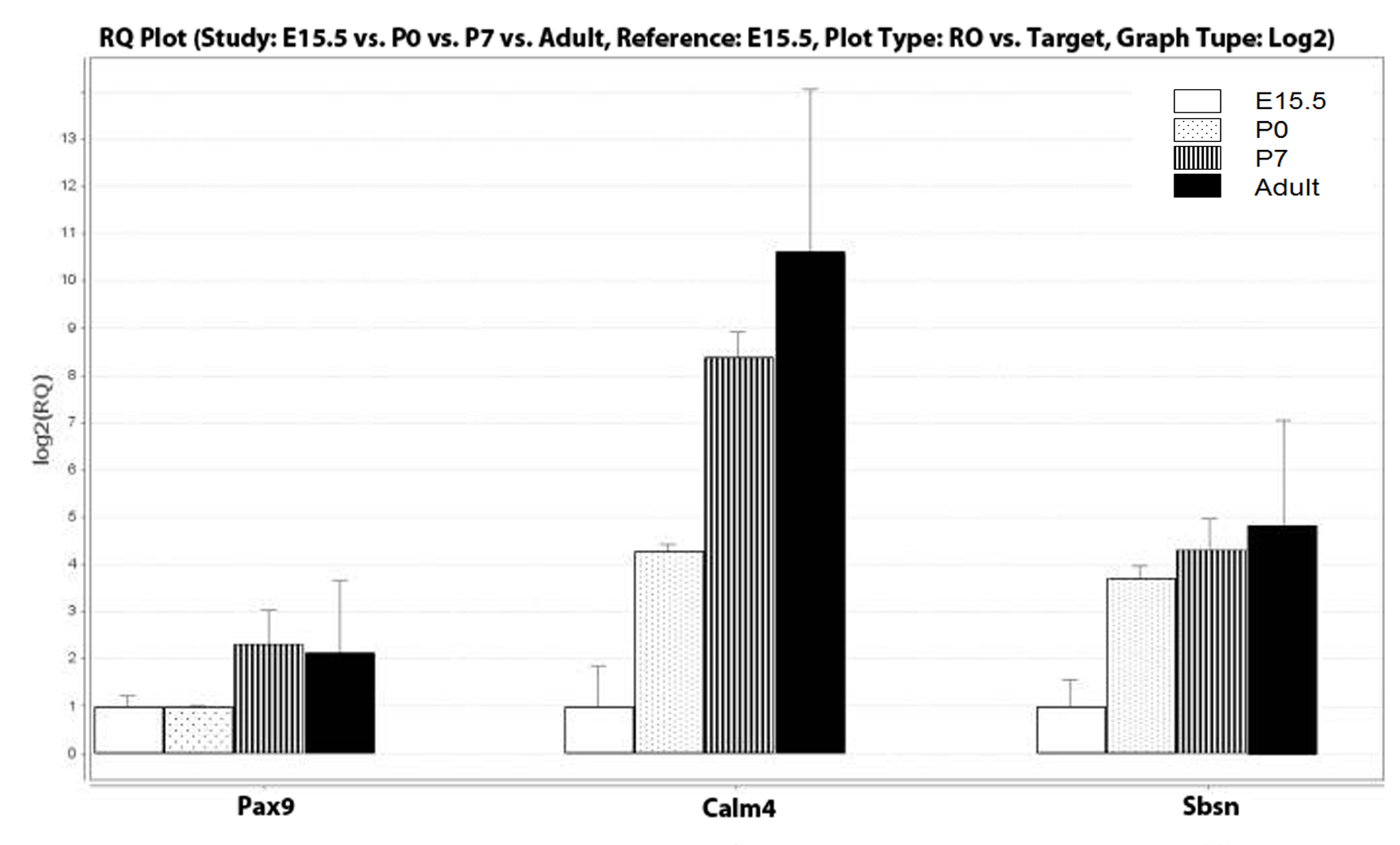

Supplement: Figure S3 — Real-time PCR analysis of mRNA expression in wild-type mouse esophagi: relative mRNA levels of Pax9 and its target genes (Sbsn, Calm4) in mouse esophageal epithelium of E15.5, P0, P7 and adult mice. (TIF) [file pone.0036504.s003.tif]

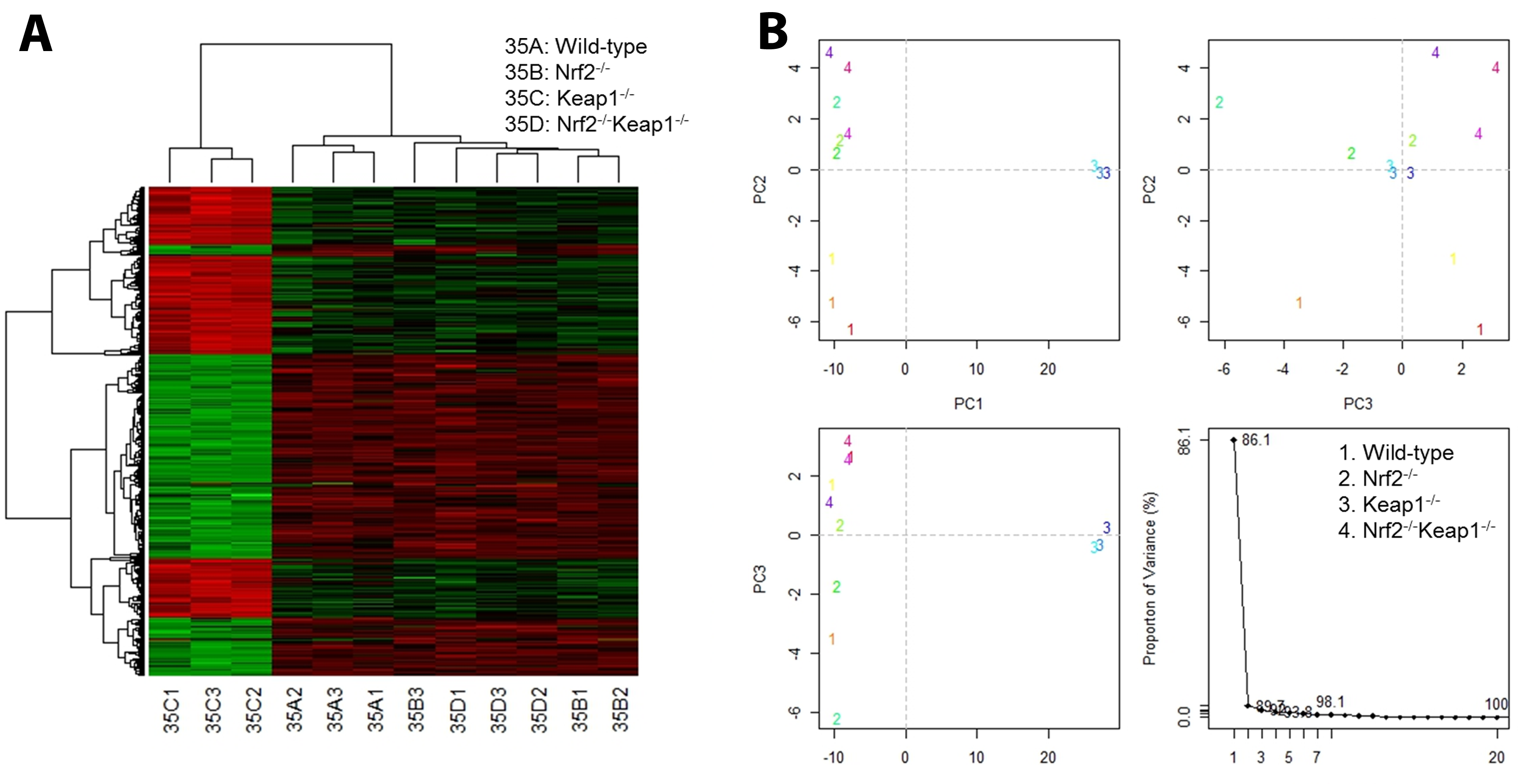

Supplement: Figure S4 — Hierarchical clustering analysis and PCA analysis of gene expression array data of P7 mouse esophagi (wild-type, Nrf2 −/−, Keap1 −/−, Nrf2 −/− Keap1 −/−): (A) clustering analysis; (B) PCA analysis. (TIF) [file pone.0036504.s004.tif]

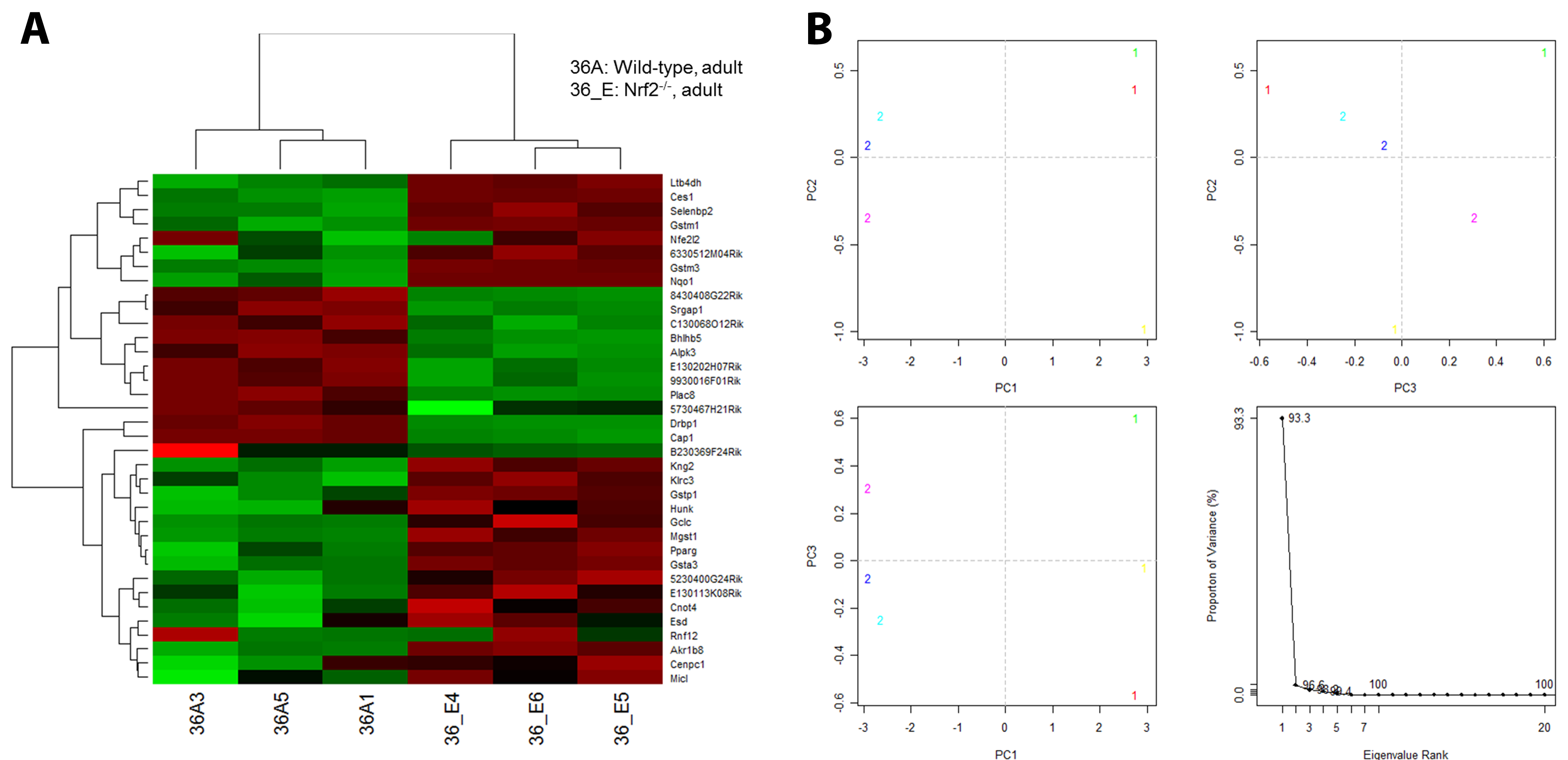

Supplement: Figure S5 — Hierarchical clustering analysis and PCA analysis of gene expression array data of mouse esophagi (wild-type adult, Nrf2 −/− adult): (A) clustering analysis; (B) PCA analysis. (TIF) [file pone.0036504.s005.tif]

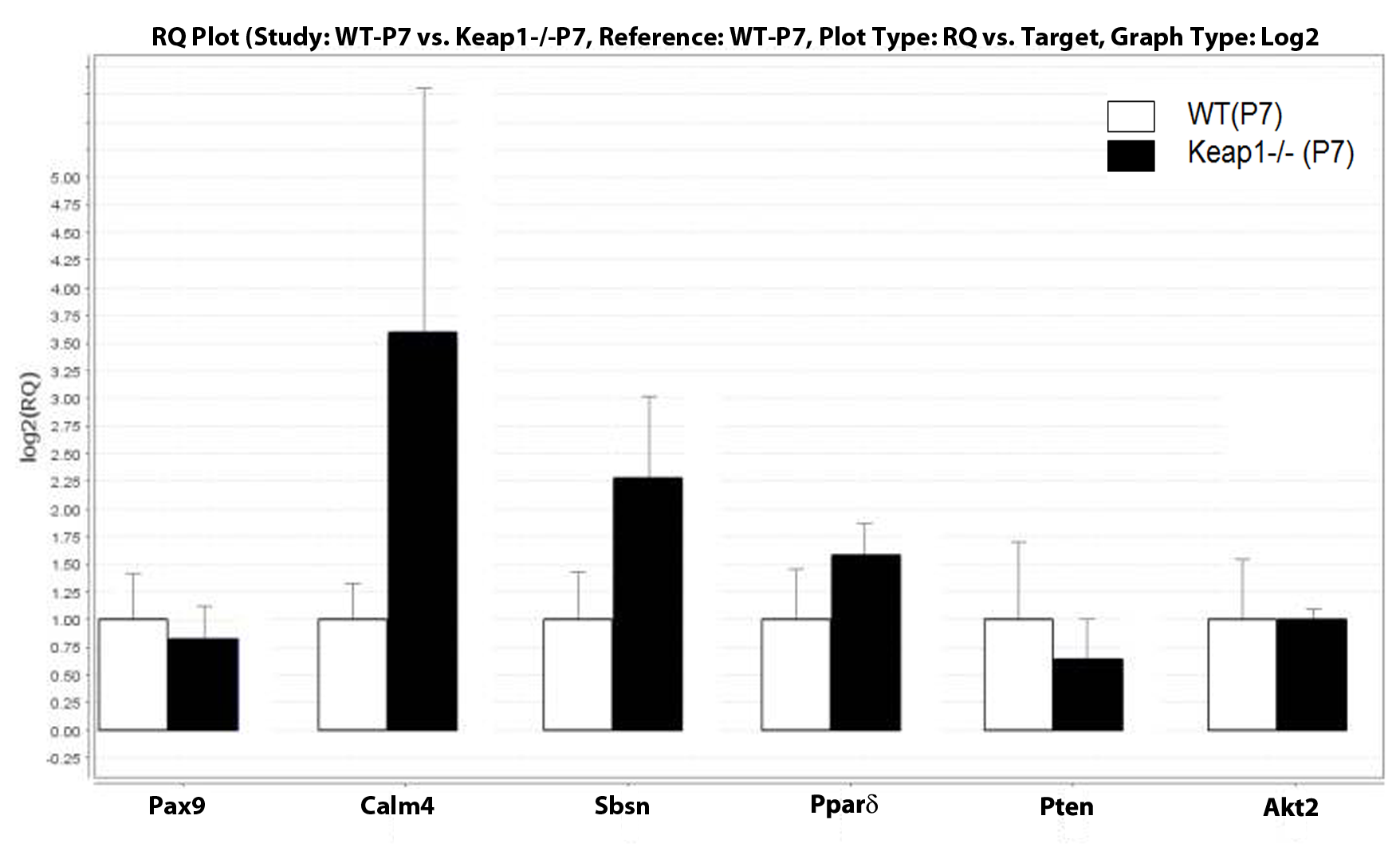

Supplement: Figure S6 — Real-time PCR analysis of mRNA expression in wild-type and Keap1 −/− mouse esophagi: relative mRNA levels of Pax9, Sbsn, Calm4, Ppard, Pten and Akt2 in the whole esophagi of wild type and Keap1 −/− mice at P7. (TIF) [file pone.0036504.s006.tif]
